# Autophagy Involvement in the Postnatal Development of the Rat Retina

**DOI:** 10.3390/cells10010177

**Published:** 2021-01-17

**Authors:** Noemi Anna Pesce, Alessio Canovai, Emma Lardner, Maurizio Cammalleri, Anders Kvanta, Helder André, Massimo Dal Monte

**Affiliations:** 1 Department of Clinical Neuroscience, Division of Eye and Vision, St. Erik Eye Hospital, Karolinska Institutet, Eugeniavägen 12, 17164 Solna, Sweden; n.pesce@student.unisi.it (N.A.P.); emma.lardner@sll.se (E.L.); anders.kvanta@ki.se (A.K.); 2 Department of Biology, University of Pisa, Via San Zeno 31, 56127 Pisa, Italy; a.canovai@studenti.unipi.it (A.C.); maurizio.cammalleri@unipi.it (M.C.); massimo.dalmonte@unipi.it (M.D.M.)

**Keywords:** eye, retina, development, vascularization, hypoxia, autophagy

## Abstract

During retinal development, a physiologic hypoxia stimulates endothelial cell proliferation. The hypoxic milieu warrants retina vascularization and promotes the activation of several mechanisms aimed to ensure homeostasis and energy balance of both endothelial and retinal cells. Autophagy is an evolutionarily conserved catabolic system that contributes to cellular adaptation to a variety of environmental changes and stresses. In association with the physiologic hypoxia, autophagy plays a crucial role during development. Autophagy expression profile was evaluated in the developing retina from birth to post-natal day 18 of rat pups, using qPCR, western blotting and immunostaining methodologies. The rat post-partum developing retina displayed increased active autophagy during the first postnatal days, correlating to the hypoxic phase. In latter stages of development, rat retinal autophagy decreases, reaching a normalization between post-natal days 14-18, when the retina is fully vascularized and mature. Collectively, the present study elaborates on the link between hypoxia and autophagy, and contributes to further elucidate the role of autophagy during retinal development.

## 1. Introduction

The mature retina is considered one of the highest oxygen-demanding tissues in the body, with a considerable metabolic activity [1,2]. The heightened metabolic demand of the retina is supplied by a structured vascular systems, including retinal vessels and the choriocapillaris, which provide nutrients and oxygen to the inner and the outer layers of the retina respectively [3,4]. During development of the mammalian eye, the retinal vasculature undergoes considerable changes and reorganization [5]. In the early stages of embryogenesis, the interior of the eye is metabolically supplied by a transient embryonic circulatory network in the vitreous, referred to as the hyaloid system [6]. In the latter stages of development, the hyaloid vasculature regresses and concurrently is replaced by the retinal vasculature [7]. The physiologic hypoxia in uterus (O_2_ levels < 5%) drives the proliferation of retinal blood vessels from the optic nerve to the periphery [8], through vascular endothelial growth factor (VEGF)-mediated angiogenesis [9,10].

At this level, the developing retinal vasculature lacks a functional barrier, necessary to maintain homeostasis into the retina and controlling vascular permeability [11,12]. Thus, the retinal capillary endothelial cells interact with each other to create a complex network, composed of tight junctions between transmembrane and peripheral membrane proteins. In this manner, the retinal endothelial cells form an inner blood retinal barrier (BRB), which contributes to preserve neuronal environment regulating the entry of molecules from the blood into the retina [13,14]. At these critical times in developmental events, both retinal and endothelial cells (ECs) endure morphological changes and reorganization; as consequence, they require mechanisms for the degradation and recycling of obsolete cellular components [15].

Autophagy is an essential process in maintaining the normal cellular homeostasis under physiological conditions [16]; and it plays an important role in the turnover of damaged organelles, such as peroxisomes and endoplasmic reticulum, as well as in removing unnecessary aggregated or misfolded proteins [17,18]. Previous findings indicate that vascular remodeling in ocular development can be regulated by autophagy [19]; the blood vessels need autophagy to balance their bioenergetic dynamic mechanisms [20]. Moreover, autophagic mechanisms seem to have a critical role in anatomical involution of the hyaloid blood vessels [19]. Several studies have demonstrated that autophagy can be induced by physiological hypoxia, the key stimulus for retinal angiogenesis [21,22,23]. At the molecular level, hypoxia stimulates several molecules involved in different signaling pathways, including hypoxia-inducible factors (HIFs) that induce angiogenesis through VEGF; and adenosine monophosphate-activated protein kinase (AMPK), a positive regulator of autophagy [24,25]. In conditions where nutrients are scarce, such as during development, AMPK is activated by a decreased ATP/AMP ratio and leads to phosphorylation of several molecules, including Unc-51-like autophagy activating kinase (ULK)1 [26,27]. Activated ULK1 is involved in the formation of multiple protein complexes that are responsible for the initiation of autophagic mechanisms that lead to the formation of autophagosome [28,29]. Elongation and maturation of the autophagosome involve the microtubule-associated protein I light chain 3 (LC3 I) system. LC3 I is conjugated to phosphatidylethanolamine, converted to LC3 II and inserted into the autophagosome membrane [30]. The synthesis and processing of LC3 II is increased during autophagy, thus acting as a key marker of levels of autophagy in cells [31]. The cargo is selected by targeted ubiquitination and carried to the autophagosome through the binding of LC3 II with sequestosome-1 (SQSTM-1), also known as ubiquitin-binding protein p62 [32,33]. The p62 is degraded by autophagy and a decrease in its protein levels correlates with an active autophagic flux [34]. Autophagy ends with the fusion of the autophagosome with the lysosome, where the inner cargo is degraded by lysosomal hydrolases.

Considering the myriad of autophagic mechanisms, the aim of the present study was to examine the changes of expression of autophagy markers in the developing retina in postnatal rats. Due to its postnatal development and accessibility, the rat retinal vasculature warrants a bonafide model to assess vascular developmental autophagy mechanisms from birth through postnatal day (P) 18 when retinal vasculature has attained its adult pattern.

## 2. Material and Methods

### 2.1. Animals and Ethics Statements

After birth, 84 Wistar rat pups were maintained with their nursing mothers through the experimental times P7, P14 and P18 in a regulated environment (24 ± 1°C, 50 ± 5% humidity), with a 12 h light/dark cycle and provided with food and water. Rat pups were euthanized with an intraperitoneal injection of 30 mg/kg of pentobarbital. All animal protocols were in accordance with the Statement for the Use of Animals in Ophthalmic and Vision Research (ARVO), the Italian regulation for animal care (DL 116/92), and the European Communities Council Directive (86/609/EEC). Animal procedures were authorized by the Ethical Committee in Animal Experiments of the University of Pisa (permit number: 133/2019-PR, 14 February 2019).

### 2.2. Vascular Labeling

A total of 24 rat pups of different ages (birth, P7, P14 and P18; six rats for each time point) were used to prepare whole-mount and retina sections. Isolated retinas were fixed in 4% paraformaldehyde in 0.1 M phosphate buffer, pH 7.4 (PB), at room temperature for 3 h. Subsequently, retinas were washed three times (5 min per wash) in PB and incubated for 1 h at room temperature (RT) in blocking buffer (PB containing 10% donkey serum and 0.5% Triton X-100; Sigma-Aldrich, St. Louis, MO, USA) to prevent non-specific labeling. Sequentially, retinas were incubated with fluorescein-labelled isolectin B4 (1:200; Vector Laboratories, Burlingame, CA, USA) in blocking solution, at 4 °C overnight (ON). Finally, after three washes with PB, retinas were placed onto a slide, mounted and covered with a coverslip. Immunostaining was observed by a digital fluorescence microscope (Ni-E; Nikon-Europe, Amsterdam, The Netherlands) and immunofluorescent images of the retinal vasculature were acquired using a digital camera (DS-Fi1c; Nikon-Europe). The vascular area and the total area were measured using ImageJ freeware. Vascular area was reported as percentage of the total area.

### 2.3. Western Blot Analysis

Proteins were extracted from retinas of 24 rat pups at different ages (birth, P7, P14 and P18; six rats for each time point) using RIPA lysis buffer (Santa Cruz Biotechnology, Dallas, TX, USA), supplemented with phosphatase inhibitor (Sigma-Aldrich) and protease inhibitor (Roche, Mannheim, Germany) cocktails. Protein extracts were quantified by the microBCA method (Thermo Fisher Scientific, Waltam, MA, USA) and 15 µg of total proteins were separated by SDS-PAGE and transferred onto polyvinylidene difluoride (PVDF) membranes (Bio-Rad Laboratories, Hercules, CA, United States). Membranes were blocked either with 5% of skim milk in Tris-buffered saline (TBS-T; Bio-Rad Laboratories, containing 0.05% Tween-20; Sigma-Aldrich) or with 4% of Bovine Serum Albumin (BSA; Sigma-Aldrich) in TBS-T, for 1 hour at RT. Subsequently, the membranes were incubated at 4 °C ON with primary antibodies: anti-HIF-1α (1:500, rabbit polyclonal, cat. no. NB100479; Novus Biologicals, Centennial, Colorado, USA); anti-pAMPKα (Thr172, 1:500, rabbit monoclonal, cat. no. 2535S; Cell Signaling Technology); anti-AMPKα (1:1000, rabbit monoclonal, cat. no. 5832S; Cell Signaling Technology); anti-pULK1 (Ser^555^; 1:500, rabbit monoclonal, cat. no. 5869S; Cell Signaling Technology, Danvers, MA, USA); anti-ULK1 (1:1000, rabbit monoclonal, cat. no. 8054S; Cell Signaling Technology); anti-LC3 I and II (1:1000, rabbit polyclonal, cat. no. 4108S; Cell Signaling Technology); anti-p62 (1:1000, rabbit polyclonal, cat. no. ab-91526; Abcam, Cambridge, UK); and anti-β-actin (1:5000, rabbit monoclonal, cat. no. SAB5600204; Sigma-Aldrich). Secondary antibody anti-rabbit-IgG conjugated to horseradish peroxidase (1:10,000, cat. no. P044801-2; Dako, Carpinteria, CA, USA) was incubated for 1 h at RT. Following the incubation with both primary and secondary antibodies the membranes were washed with TBS-T, three times for 5 min. Finally, the protein of interest was visualized using the Clarity Western ECL substrate with a ChemiDoc XPS^+^ imaging system (Bio-Rad Laboratories, Hercules CA, USA). The optical density (OD) of the bands was determined with the Image Lab 3.0 software (Bio-Rad Laboratories). Protein levels were corrected to the β-actin loading control or non-phosphorylated proteins.

### 2.4. Quantitative PCR

Total RNA was extracted and purified from retinas of 24 rat pups at different ages (birth, P7, P14 and P18; six rats for each time point), using the Trizol^®^ reagent (Invitrogen, Waltam MA, USA), resuspended in RNAse-free water, and quantified by spectrophotometry (BioSpectrometer basic; Eppendorf AG, Hamburg, Germany). First-strand cDNA was generated from 1 μg of total RNA (QuantiTect Reverse Transcription Kit; Qiagen, Hilden, Germany). Quantitative PCR was performed with a kit (SsoAdvanced Universal SYBR Green Supermix; Bio-Rad Laboratories) on a CFX96 Real Time PCR Detection System (equipped with the CFX manager software (Bio-Rad Laboratories). Forward and reverse sequence of primers were chosen to hybridize to unique region of the appropriate gene sequence: occludin-1 (Forward: 5’-TTTCATGCCTTGGGGATTGAG-3’/Reverse: 5’-GACTTCCCAGAGTGCAGAGT-3’; Invitrogen ); zonula-occludens-1 (ZO-1; Forward: 5’-AGTCTCGGAAAAGTGCCAGG-3’/Reverse: 5’-GGGCACCATACCAACCATCA-3′; Invitrogen); VEGF-A (Forward: 5’-CAATGATGAAGCCCTGGAGTG-3’/Reverse: 5’-AGGTTTGATCCGCATGATCTG-3; Invitrogen) and Ribosomal Protein L13a (Rpl13a; Forward: 5′-GGATCCCTCCACCCTATGACA-3′/Reverse: 5′-CTGGTACTTCCACCCGACCTC-3′; Invitrogen). Samples were compared by the threshold cycle analysis (Ct) and absolute expression values were calculated using the 2^−ΔΔCt^ formula, with Rpl13a as the housekeeping gene.

### 2.5. Immunohistofluorescence

In total, 12 rat pups at different ages (birth, P7, P14 and P18; three rats for each time point) were fixed in 4% paraformaldehyde in PB at RT for 5 days prior to immunofluorescence staining. Sections of 4 micrometers were processed for immunohistofluorescence in a Bond III robotic system (Leica Biosystems, Newcastle, UK), as previously described [35]. According to the manufacturer’s instructions, antigen retrieval was performed in 10 mM citrate buffer pH6 and sections were incubated with primary antibody step with anti-LC3 I and II (1:100) and isolectin-biotin (1:1000; cat. no. I21414; Thermo Fisher Scientific), while secondary antibody steps included Streptavidin-Alexa 488 (1:500; cat. no. S11223, Thermo Fisher Scientific), anti-rat-Alexa 546 (1:500; cat. no. A11081, Invitrogen) and anti-goat-Alexa 647 (1:500; cat. no. SAB4600175, Sigma-Aldrich). Sections were mounted by using a vector Vectashield with DAPI mounting medium (Vector Laboratories, CA, Burlingame, USA), and visualized with an Axioscope 2 plus with the AxioVision software (Zeiss, Gottingen, Germany).

### 2.6. Statistical Analysis

The statistical analyses were performed using Prism software (GraphPad software, Inc., San Diego, CA, USA), applying one-way ANOVA with Bonferroni’s multiple comparisons posttest. Data were presented as mean ± standard error of mean (SEM) of *n* = 6; *p* values < 0.05 were considered statistically significant.

## 3. Results

### 3.1. The Rat Retina Is Partially Avascular at Birth

**Figure 1:**
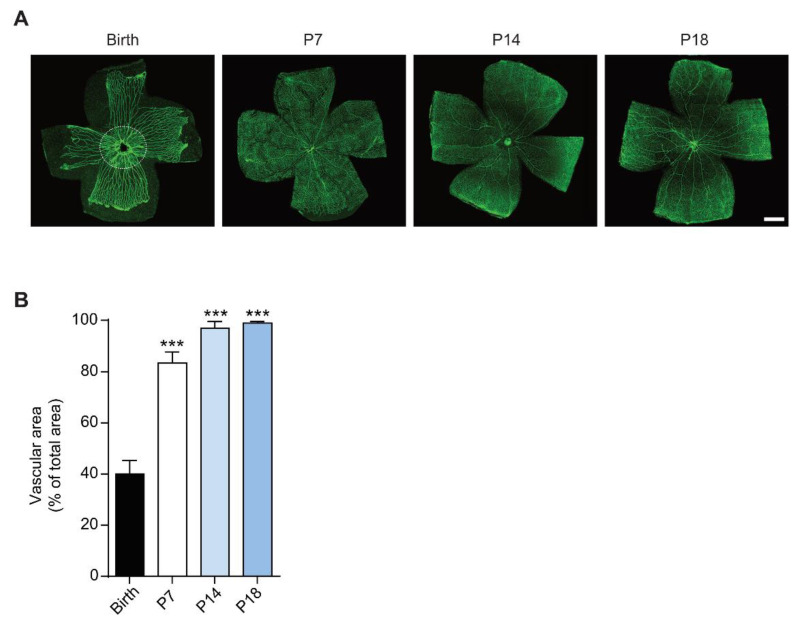
Development of the vascular network in rat retina. (**A**) Visualization of blood vessels by isolectin B4 staining of rat pup retinas at birth, postnatal day (P)7, P14 and P18. Dashed circle delineates the developing retinal vasculature (inner of the dashed circle) with the hyaloid vasculature (outer of the dashed circle). Scale bar = 500 μm. (**B**) Quantitative analysis of vascular area of the retina. Data is presented as mean ± SEM. One-way ANOVA was used as statistical analysis, followed by Bonferroni’s multiple comparisons test (*n* = 6; *** *p* < 0.001 vs birth).

Fluorescein-labeled isolectin B4 was used to assess the progress of retinal vascularization in the rat. As previously described, rat retinal vascularization is almost completed around P13-P16 [36]. As depicted in Figure 1A, at birth the hyaloid vasculature was still present and the retina remained partially avascular, as determined by measuring the vascular area as less than 50% of total area of the retina (Figure 1B). Quantitative analysis demonstrated a significant difference at P7, where the retina was 90% vascularized (*p* < 0.001) and in the late stages of ocular blood vessel development, P14-P18 (*p* < 0.001), where the retinas were fully vascularized (100%) as compared to birth.

### 3.2. Different Expression of Blood-Retina Barrier Genes during Rat Retina Development

**Figure 2:**
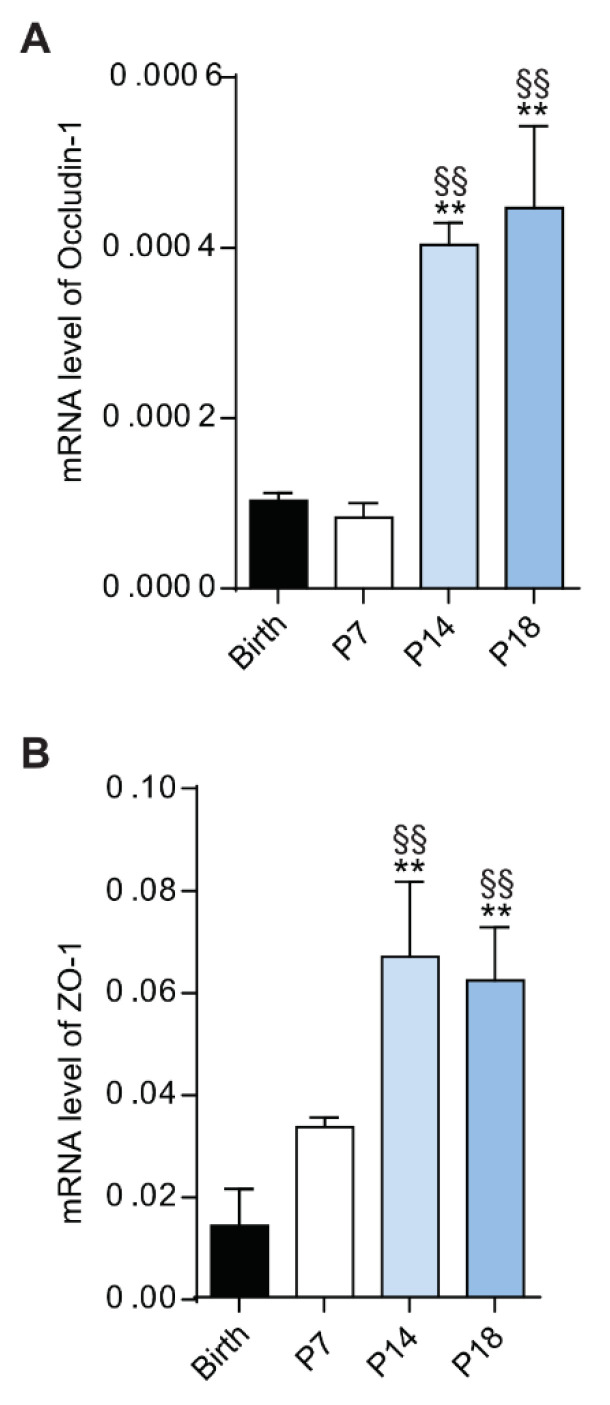
Expression of blood-retina barrier genes during retina rat development. mRNA expression of occludin-1 (**A**) and zonula occludens (ZO-1) (**B**) genes was evaluated by qPCR in rat retinas at birth, P7, P14 and P18. Data is presented as mean ± SEM. One-way ANOVA was used as statistical analysis, followed by Bonferroni’s multiple comparisons test (*n* = 6; ** *p* < 0.01 vs birth, ^§§^ *p* < 0.01 vs P7).

In retinal blood vessels, ECs present tight junctions that function as a part of the BRB, fundamental to maintain retinal homeostasis and to mediate selective diffusion of molecules from the circulation to the retinal tissue [14,37]. Consequently, analysis of the expression of BRB genes in the rat retinas at birth, P7, P14 and P18 was performed by qPCR (Figure 2). Transcript expression levels of occludin-1 and ZO-1 were upregulated at P14 and P18, compared to birth and P7 (*p* < 0.01 both). These results confirmed that at birth and P7 the rat retinal vasculature was still under development, presenting an incomplete vascular network.

### 3.3. At P7 the Rat Retina Is Hypoxic

**Figure 3:**
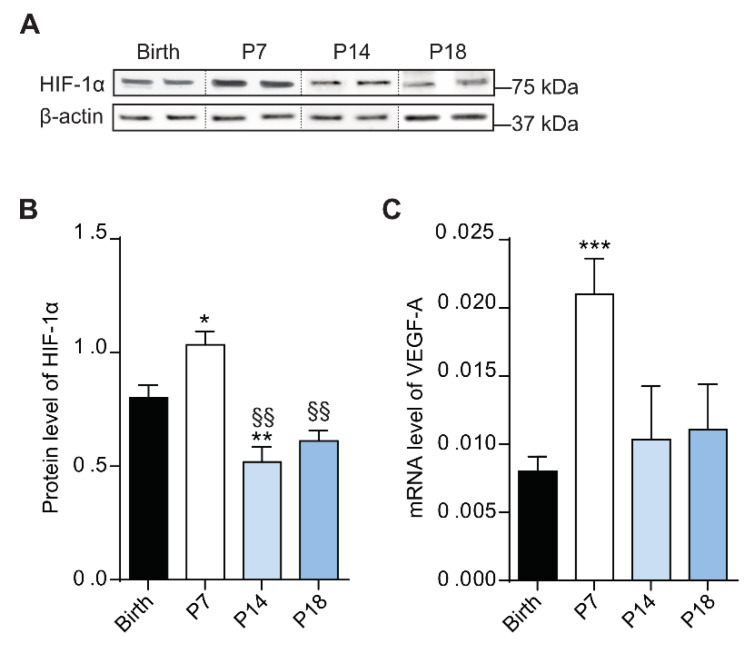
Protein levels of hypoxia-inducible factor (HIF) change during rat retina development. (**A**) Western blots illustrate representative immunoreactive bands of HIF-1α and β-actin (loading control) in the retina of rat pups from birth to P18. (**B**) Quantitative analysis of optical density of the immunoreactive bands of HIF-1α. (**C**) mRNA expression of vascular endothelial growth factor (*VEGF)-A* gene evaluated with qPCR in rat retinas at birth, P7, P14 and P18. One-way ANOVA followed by Bonferroni’s multiple comparisons test was used as statistical analysis of mean ± SEM datasets (*n* = 6; * *p* < 0.05, ** *p* < 0.01, *** *p* < 0.001 vs birth, ^§§^ *p* < 0.01 vs P7).

During retinal development, physiologic hypoxia induces the activation of HIF-1α, which promotes the transcription of *VEGF-A* gene. This process is pivotal to induce endothelial cell proliferation and migration to form the vasculature network [38]. In this context, Western blotting was performed to analyze HIF-1α protein levels in the rat retina (Figure 3A) at birth, P7, P14 and P18. Densitometric analysis showed an increase of HIF-1α protein levels at P7 (*p* < 0.001) compared to birth, followed by a decrease at P14 (*p* < 0.01 vs birth; *p* < 0.01 vs P7) and at P18 (*p* < 0.01 vs P7; Figure 3B).

Since HIF-1α promotes the transcription of *VEGF-A* gene, expression analysis was performed by qPCR in rat retinas at birth, P7, P14 and P18. As depicted in Figure 3C, VEGF-A was upregulated at P7, (*p* < 0.001 vs birth) while at P14 and P18 its levels were comparable to those at birth, in agreement with the reduced levels of HIF-1α at the latter postnatal days.

### 3.4. Autophagic Mechanisms Increased during the Hypoxic Phase

**Figure 4:**
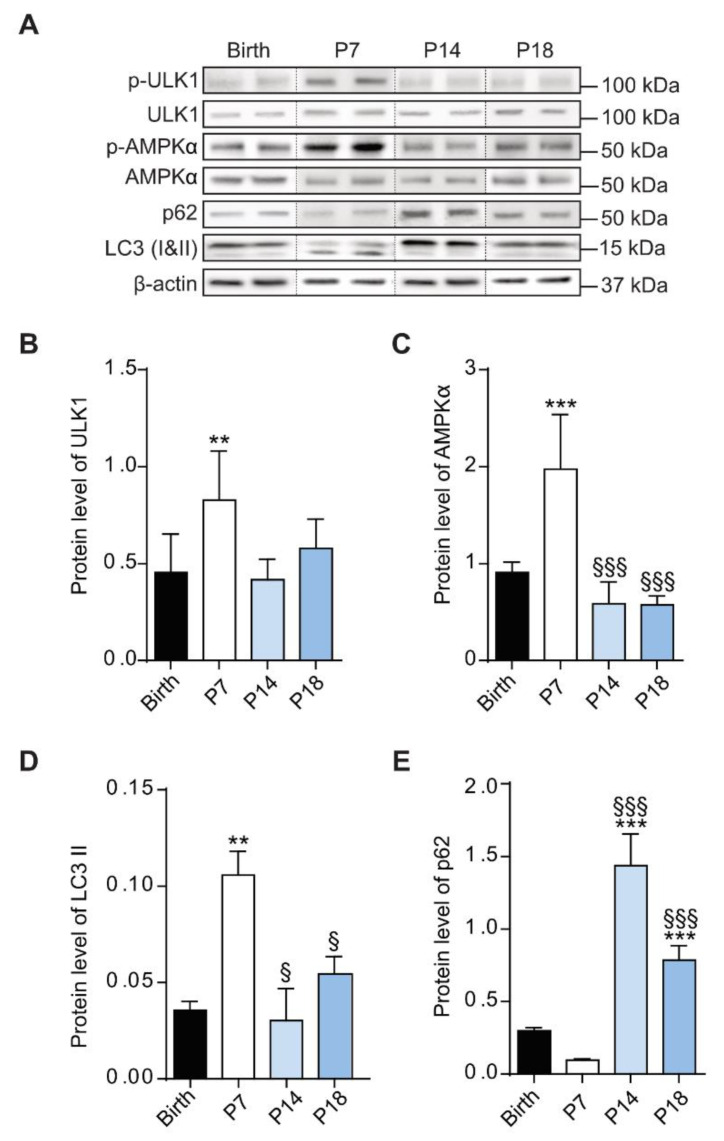
Protein levels of autophagic markers during rat retinal development. (**A**) Western blots depict representative immunoreactive bands of proteins involved in autophagic mechanisms in rat retinas, from birth to P18. Quantitative analysis of optical density of the ratio of immunoreactive bands between pSer^555^-Ulk1 / Ulk1 (**B**), *p*-AMPKα / AMPKα (**C**); LC3 II / β-actin (**D**) and p62 / β-actin (**E**). One-way ANOVA followed by Bonferroni’s multiple comparisons test was used as statistical analysis of mean ± SEM datasets (*n* = 6; ** *p* < 0.01, *** *p* < 0.001 vs birth, ^§^ *p* < 0.05, ^§§§^ *p* < 0.001 vs P7).

During development, autophagic mechanisms support cells to adapt and respond to several processes, including proliferation, differentiation and migration [39]. In this context, Western blotting analysis was performed to evaluate the levels of proteins involved in autophagy during retina development (Figure 4A). Densitometric analysis demonstrated a significant increase of phosphorylated levels of AMPKα (*p* < 0.001 vs birth; Figure 4C) and ULK1 (*p* < 0.01 vs birth; Figure 4B) as well as LC3 II (*p* < 0.01 vs birth; Figure 4D) at P7. At P14 and P18, the levels of these autophagic markers decreased then at levels comparable to those at birth.

On the contrary, a trend to a decrease of SQSTM1/p62 protein levels, yet not statistically significative, was observed at P7, followed by a substantial increase at both P14 (*p* < 0.001 vs birth; *p* < 0.001 vs P7) and P18 (*p* < 0.001 vs birth; *p* < 0.001 vs p7).

### 3.5. High Expression of Autophagic Marker at P7 in Rat Retina

**Figure 5:**
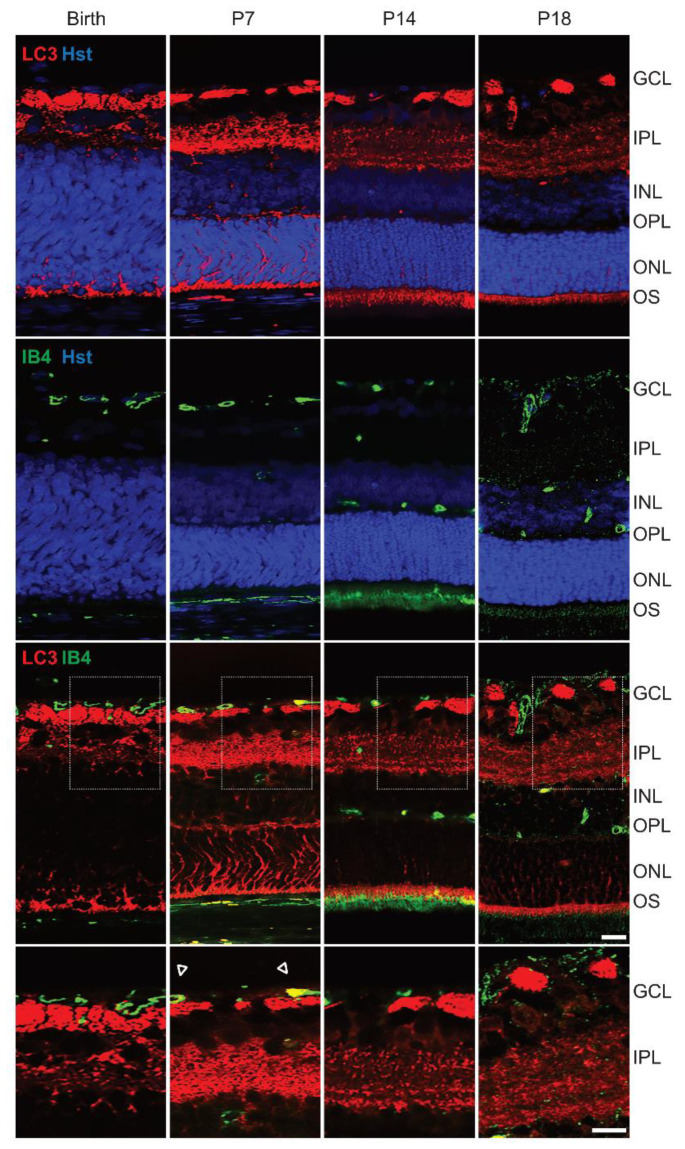
Expression pattern of LC3 in the developing rat retina. Representative immunohistofluorescence analysis of LC3 (red) and isolectin B4 (IB4; green) and Hoechst (Hst; blue) in retina sections of rat pups at birth, P7, P14 and P18. Dashed squares indicate the magnification area of GCL and IPL layers. Arrows represent colocalization of LC3 with IB4 in retinal vasculature. GCL, ganglion cell layer; IPL, inner plexiform layer; INL, inner nuclear layer; OPL, outer plexiform layer; ONL, outer nuclear layer; OS, outer segments of photoreceptors. Scale bar = 50 μm.

In rat, developing retinal cells are already organized into layers from P7, giving the tissue its stratified feature [40,41]. The variation of autophagic flux was evaluated relative to the retinal layers using immunostained retinal sections to visualize autophagy and vasculature, with LC3 as an autophagic marker and isolectin B4 as a marker of endothelial cells. The immunofluorescence demonstrated a clear variation of autophagy during retinal development, indicating a predominant expression of the autophagic marker LC3 at P7 (Figure 5). At this specific time point, a substantial expression of LC3 was observed in both the inner plexiform and outer plexiform layers (IPL; OPL). At birth and on latter stages of retinal developmental, LC3 was predominantly expressed in the IPL and almost undetectable in the OPL. In addition, heightened colocalized expression of the autophagy and vascular markers was observed at P7, as compared to birth, P14 and P18, which could be related to the hypoxia associated with the involution of the hyaloid blood vessels. Albeit a positive staining for LC3 was detected in ganglion cell layer (GCL) and the photoreceptor outer segments (OS), no changes were observed in the studied times of development.

## 4. Discussion

During mammalian development, cells go through proliferation, cell death and differentiation, culminating in an adult organism formation. During these stages, autophagy assures cell adaptation, by promoting rapid changes in cytosolic composition and accelerating organelle and protein turnover [39,42]. The present study elaborates on the influence of autophagy mechanisms in the development of rat’s retina from birth to P18.

In rats, the retinal vasculature develops postnatally. At birth, the retina surface is still covered by the hyaloid vessels, and the retinal vessels have merely begun to raise from the optic disc [43]. Previous studies have shown that the hyaloid network persists until P7, by which time the retinal vessels have almost propagated to the periphery of the retina [44,45]. Interestingly, in newborn rat retinas a large area lacking retinal capillary coverage is observed, indicating the presence of hyaloid vessels, while at P7 the rat retinas display nearly full vascularization. At this time point, the developing retina vascular network is still immature and the BRB is not formed [11]. To form the BRB, endothelial cells require tight junctions, essential to regulate the movement of solutes and nutrients from the outer to the inner retinal layers [46]. Tight junctions comprise several proteins, including occludin-1 and ZO-1, responsible for anchoring the junctional complex to the cytoskeleton [47]. As demonstrated here, at birth and P7 the retina of rat pups presents low transcript levels of both occludin-1 and ZO-1 genes. Reversely, mRNA levels of these genes are increased at P14 and P18, when the retinal vasculature is covering the total surface of retina. This suggests that retina vascularization is completed and mature around P14-P18, in alignment with the presence of tight junctions in retinal endothelial cells.

From embryonic stages to birth, a physiological hypoxia is paramount to drive retinal neovascularization, through the upregulation of HIF-1α and subsequently VEGF-A [9,48]. During the early postpartum retinal developmental stage in rats, the involution of the hyaloid vasculature to the retinal vascular network results in a partially avascular and ischemic retina, correlating to increased oxygen demand and resulting in a hypoxic stimulus [10]. In the present study, a peak of HIF-1α and VEGF-A expression is determined at P7, which decreases during latter stages when the retina is fully vascularized. The hypoxic environment contributes to activate essential mechanisms in adaptation and survival, ensuring cellular homeostasis during angiogenesis [49,50]. In fact, the developing retina exposed to changing environment and metabolic stress requires autophagy to adjust its bioenergetic and biosynthetic demands [20,49]. In this respect, both hypoxia and energy deprivation can promote AMPK activation, a known inducer of autophagy [22,25]. In agreement, an increase of p-AMPKα, p-ULK1 and LC3 II protein levels are demonstrated at P7, with a trend of decreased p62 protein levels, indicating an active autophagic flux at this developmental stage. At P14 and P18, with the presence of a fully vascularized retina, a decrease in p-ULK1 and LC3 II protein levels is determined concomitantly with an increase of p62. The observed accumulation of p62 with a decrease of LC3 II protein levels at P14 could be associated with a transition from autophagy-dependent to -independent mechanisms of retinal homeostasis, as previously suggested in retinal pigment epithelium cells [51,52].

To elaborate on the role of autophagy in the different cell layers during rat retinal development, a predominant expression of LC3 is confirmed in the GCL, IPL, OPL and OS, in agreement with previous studies in rodent retinas [53,54,55]. At P7, an increase of LC3 staining is denoted in the IPL and OPL, with a noticeable reduction in the autophagy marker at P14 and P18. During the early postpartum developmental stage, the rat retinal cells are affected by ischemia, which is correlated to the peak of expression of HIF-1α and VEGF-A, concomitantly with LC3. Moreover, an expression of LC3 is observed in endothelial cells at P7, suggesting that autophagy may contribute directly to the formation of the retinal vascular network. These findings indicate that during the physiologic hypoxia in the rat retina, HIF-mediated signaling induces the increase in VEGF-A to promote endothelial cell proliferation, and an upregulation of autophagy markers to sustain cellular homeostasis and cellular quality control in retinal cells.

## 5. Conclusions

The present study indicates that increased autophagy is intrinsically associated with the hypoxic phase of retinal development and critically contributes to the physiologic development of the different cell layers of the retina during the transition from the hyaloid to the retinal vasculature, thus allowing the normal development of the retina.

## Data Availability

The data presented in this study are available on request from the corresponding author.
